# Glucose transporters in adipose tissue, liver, and skeletal muscle in metabolic health and disease

**DOI:** 10.1007/s00424-020-02417-x

**Published:** 2020-06-26

**Authors:** Alexandra Chadt, Hadi Al-Hasani

**Affiliations:** 1 Medical Faculty, Institute for Clinical Biochemistry and Pathobiochemistry, German Diabetes Center, Leibniz Center for Diabetes Research at Heinrich Heine University Düsseldorf, Auf’m Hennekamp 65, 40225 Düsseldorf, Germany; 2 German Center for Diabetes Research (DZD), Munich-Neuherberg, Germany

**Keywords:** Crosstalk, Exercise, Insulin resistance, NAFLD, Type 2 diabetes

## Abstract

A family of facilitative glucose transporters (GLUTs) is involved in regulating tissue-specific glucose uptake and metabolism in the liver, skeletal muscle, and adipose tissue to ensure homeostatic control of blood glucose levels. Reduced glucose transport activity results in aberrant use of energy substrates and is associated with insulin resistance and type 2 diabetes. It is well established that GLUT2, the main regulator of hepatic hexose flux, and GLUT4, the workhorse in insulin- and contraction-stimulated glucose uptake in skeletal muscle, are critical contributors in the control of whole-body glycemia. However, the molecular mechanism how insulin controls glucose transport across membranes and its relation to impaired glycemic control in type 2 diabetes remains not sufficiently understood. An array of circulating metabolites and hormone-like molecules and potential supplementary glucose transporters play roles in fine-tuning glucose flux between the different organs in response to an altered energy demand.

## Introduction

**Fig. 1:**
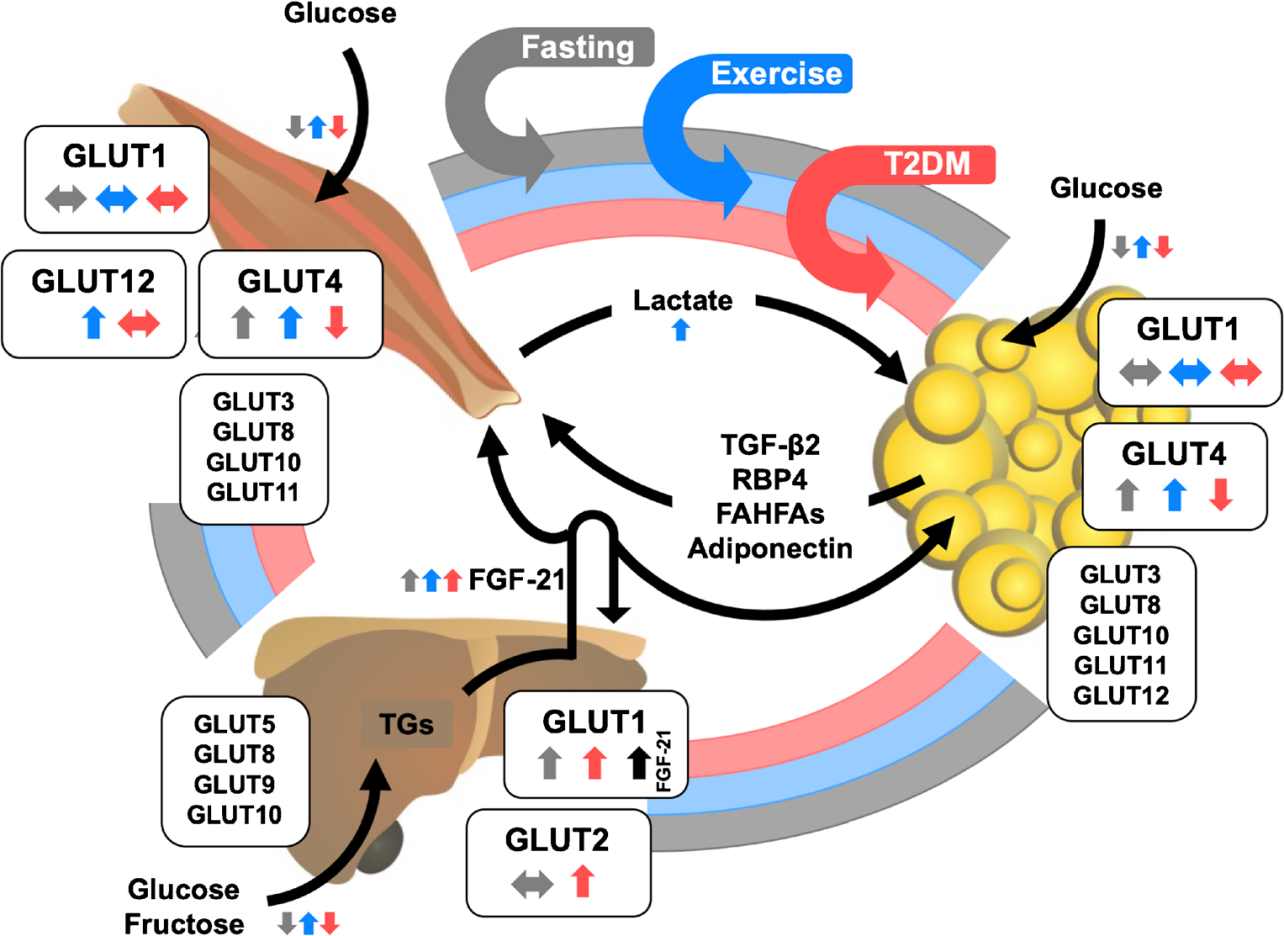
Integrative physiology of glucose transporters (GLUTs) in the liver, skeletal muscle, and adipose tissue. Expression levels of main GLUT isoforms are regulated by a diversity of metabolic stimuli including fasting and physical activity (exercise) and by certain pathophysiological conditions such as type 2 diabetes (T2DM). A complex inter-organ network is necessary to maintain whole-body energy metabolism in balance. This interaction is regulated by secretion of various factors into the circulation to facilitate tissue crosstalk. The distinct trigger mechanisms for the secretion of these factors are indicated by the respective arrow color (gray, fasting conditions; blue, exercise/physical activity; red, T2DM). In addition, the impact of these three (patho)physiological conditions on gene and/or protein expression of the diverse GLUTs as well as transport of GLUT substrates (e.g., glucose, fructose) is presented by small colored arrows next to the respective GLUT. TGs, triglycerides; FGF-21, fibroblast growth factor 21; TGF-β2, transforming growth factor β2; RBP4, retinol binding protein 4; FAHFAs, fatty acid esters of hydroxy fatty acids

Glucose represents the major source of energy for most tissues of the body. Thus, maintenance of whole-body glucose homeostasis is the result of a complex regulatory system involving various tissues. Inter-organ crosstalk via a diversity of circulating factors such as hormones and neuropeptides ensures distribution of nutritional components according to the respective need of the specific organ [84]. At present, three classes of eukaryotic sugar transporters have been characterized: glucose transporters (GLUTs) belonging to the *SLC2A* gene family, sodium-glucose symporters (SGLTs), and SWEETs [32]. The large family of GLUTs, evolutionary conserved facilitative glucose transporters, is involved in all critical steps of handling glucose and other hexoses, including absorption, distribution, and excretion/recovery. Intake of carbohydrates leads to an immediate increase in circulating blood glucose levels after absorption of the glucose from the intestine. As a direct response, pancreatic beta cells sense the elevated blood glucose concentrations via a GLUT2-dependent process and increase secretion of insulin. Consequently, insulin binding to its receptors leads to enhanced glucose transport into skeletal muscle, adipose tissue, and the heart, mainly facilitated by an acute translocation of GLUT4 transporter vesicles to the plasma membrane and, in addition, to an inhibition of hepatic gluconeogenesis. Both regulatory pathways in combination result in the clearance of glucose from the bloodstream. Insulin resistance represents a state of relative unresponsiveness of peripheral tissues to react accordingly to increasing amounts of insulin in the circulation, resulting in chronically elevated blood glucose levels. This state of hyperglycemia is known to be a hallmark of type 2 diabetes mellitus, a major health burden of modern society, characterized by a progressive increase in peripheral insulin resistance followed by beta cell destruction and, as a result, hypoinsulinemia. The pathophysiology of this metabolic disease is not yet completely understood; however, there is strong evidence for a crucial role of different members of the GLUT family during development and progression of insulin resistance and type 2 diabetes (Fig. 1).

This article highlights the function of the GLUT family in the liver, muscle, and fat tissue and the specific contribution of GLUTs to systemic glucose homeostasis and energy metabolism in the healthy and diabetic state. Other recent reviews provide excellent and thorough overviews on the structure/function relationship of GLUTs [32, 93, 260], insulin signaling [83], and the regulation of the insulin- and contraction-responsive GLUT4 trafficking [100, 118]. Table 1 summarizes the tissue-specific function of the GLUTs in metabolism.

**Table 1:** Overview of main GLUTs in the liver, muscle, and adipose tissue and their tissue-specific function in metabolism

Tissue	Isoform	Tissue-specific function in metabolism
Liver	GLUT1	Postnatal development and organogenesis of the liver [89]; main glucose transporter in non-parenchymal cells, relatively low levels in hepatocytes [221]; elevated in non-alcoholic steatohepatitis (NASH), alcoholic liver disease (ALD) [109], and hepatocellular carcinoma (HCC) [267]; reduced surface expression in hepatitis C virus (HCV) infection [111]; may contribute to glucotoxicity and oxidative stress [220]
	GLUT2	Most abundant GLUT isoform in hepatocytes, responsible for bulk of glucose uptake, but does not directly mediate hepatic glucose output [80]; involved as hepatoportal glucose sensor [20, 21]; *SLC2A2* deficiency causal for Fanconi–Bickel syndrome (FBS) [61, 144]; gene variants have been associated with fasting hyperglycemia, transition to type 2 diabetes, hypercholesterolemia, and the risk of cardiovascular diseases [60]; downregulated in HCV infection [111]
	GLUT5	Fructose transport, dietary fructose consumption associated with increased expression, non-alcoholic fatty liver disease (NAFLD) [10]
	GLUT8	Mediates fructose-induced de novo lipogenesis [44]; overexpression linked to decreased PPARγ expression levels [43]; expression correlates with circulating insulin in diabetic mice [77]; involved in trehalose-induced autophagy [150]
	GLUT9	High-capacity uric acid (UA) transporter; hepatic inactivation of the gene in adult mice leads to severe hyperuricemia and hyperuricosuria [177]
Muscle	GLUT1	Contributes to basal glucose transport and fiber type–specific expression [106, 146]; increased surface expression in metabolic stress [195, 216]; increased overload-induced muscle glucose uptake or hypertrophic growth [153]
	GLUT4	Most abundant GLUT isoform, responsible for bulk of insulin- and contraction-stimulated glucose uptake [50, 131, 148]; insulin/contraction-regulated subcellular distribution between intracellular compartments and cell surface [38, 58, 67, 229]; knockout mice display systemic insulin resistance and a mild diabetic phenotype [115]; overexpression improves insulin sensitivity [19, 237]; upregulated in response to exercise [185]; abundance in diabetic skeletal muscle is mostly unchanged [174]
	GLUT10	Localized in mitochondria, involved in mitochondrial dehydroascorbic acid (DHA) transport, may protect from oxidative stress [126]; increased in overload-induced muscle glucose uptake or hypertrophic growth [153]
	GLUT12	May act as insulin-responsive glucose transporter similar to GLUT4 [225]; upregulated in humans after intensive exercise training [224]
Adipose	GLUT1	Contributes to basal glucose transport, undergoes recycling through internal membrane compartments [94]; abundance unaffected in type 2 diabetes [105]
	GLUT8	Expression increases markedly during fat cell differentiation [206]; recycles between endosomal compartments and cell surface, mostly intracellular, in mature adipocytes unresponsive to insulin [9, 128]
	GLUT4	Most abundant GLUT isoform, responsible for bulk of insulin stimulated glucose uptake [104]; activity associated with activation of nuclear transcription factor carbohydrate-response element-binding protein (ChREBP), enhanced lipogenesis and production of branched fatty acid esters of hydroxy fatty acids (FAHFAs) and secretion of retinol binding protein 4 (RBP4) [91, 160, 261]; reduced abundance in type 2 diabetes [69, 219]
	GLUT10	Mitochondrial DHA transport, may protect from oxidative stress [126]

## The liver

### The liver is the main organ for glucose storage and essential for the regulation of glucose homeostasis

The liver represents one of the most crucial organs in the regulation of whole-body glycemia. In addition to its important role in energy storage, mainly as glycogen and triglycerides, it has the unique function to export glucose in times of energy demand. Triggered by low glucose levels during starvation or in between meals, the peptide hormone glucagon is secreted from pancreatic alpha cells, stimulating the breakdown of glycogen to glucose molecules (glycogenolysis) and the production of glucose from non-carbohydrate precursors such as glucogenic amino acids or pyruvate during de novo glucose production (gluconeogenesis) in the liver. Both pathways enable the liver to provide appropriate amounts of glucose for all other organs, specifically the brain, an organ heavily relying on glucose as main fuel source. In contrast, postprandial hyperglycemia and hyperinsulinemia result in the stimulation of hepatic glycogen synthesis, on the one hand, and inhibition of gluconeogenesis, on the other hand [196]. In the healthy state, physiological hyperinsulinemia has been demonstrated to completely suppress hepatic glycogenolysis while gluconeogenesis is reduced by 20% [70]. The hepatic glucose production (HGP) is comprised of the processes of glycogenolysis and gluconeogenesis. A postprandial elevation of blood glucose concentration leads to the enhanced secretion of insulin from pancreatic beta cells, acting on the liver both directly and indirectly. Direct effects of insulin on HGP are mediated by binding of insulin to the respective tyrosine kinase receptors on the cell membrane, subsequently inhibiting glycogenolysis by facilitating suppression of glucose-6-phosphatase activity and several enzymes involved in glycogen synthesis, including phosphofructokinase and glycogen synthase [173]. Whereas the exact mechanisms behind direct insulin-mediated regulation of hepatic gluconeogenesis are unclear, several indirect regulatory pathways have been demonstrated. The indirect control of insulin on HGP involves several mechanisms and diverse other organs. Insulin-mediated inhibition of lipolysis in the adipose tissue results in reduced levels of circulating free fatty acids and glycerol. In addition, glucagon production is inhibited by insulin in pancreatic alpha cells. Both processes consequentially lead to decreased hepatic glucose output in the postprandial state, maintaining normoglycemia [33, 218].

### Liver insulin resistance is a major feature of type 2 diabetes pathophysiology

Hepatic insulin resistance has been characterized by a reduction of insulin-stimulated signal transduction pathways for hepatic glucose production, including insulin receptors and downstream mediators [175]. Several factors are known to be causative for the development of insulin resistance in the liver. For instance, infections with hepatitis C virus (HCV) are strongly associated with the progression of hepatic insulin resistance and type 2 diabetes occurrence. Mechanistically, HCV core protein leads to upregulation of inflammatory markers such as tumor necrosis factor α (TNF-α), eventually resulting in reduced downstream activation of insulin signaling [36]. In addition, HCV core protein triggers oxidative stress in hepatocytes by causing dysfunction at the mitochondria and the endoplasmic reticulum (ER), promoting triglyceride accumulation and liver steatosis [212]. A tight relationship exists between various chronic metabolic diseases, such as obesity, type 2 diabetes, and non-alcoholic fatty liver disease (NAFLD), all of them reaching epidemic dimensions on a global scale [258]. While NAFLD increases type 2 diabetes incidence and the occurrence of late complications, type 2 diabetes accelerates NAFLD progression towards even more fatal liver disorders such as cirrhosis, hepatocellular carcinoma, and non-alcoholic steatohepatitis (NASH) [248]. Importantly, NAFLD can be considered as a reliable predictor for the development of type 2 diabetes [12]. In general, high concentrations of lipids and specific lipid derivates such as ceramides or diacylglycerols (DAGs)—a characteristic feature of NAFLD and NASH—are known to exert toxic effects on liver cells, a process referred to as “lipotoxicity.” In addition, chronic hyperglycemia and excess carbohydrate influx into the liver are associated with the accumulation of hepatotoxic lipids as well. This “glucotoxicity” also includes the activation of lipogenic enzymes and induction of ER stress, eventually resulting in steatosis and cell death [162].

### Several members of the GLUT family are relevant in liver metabolism

**Fig. 2:**
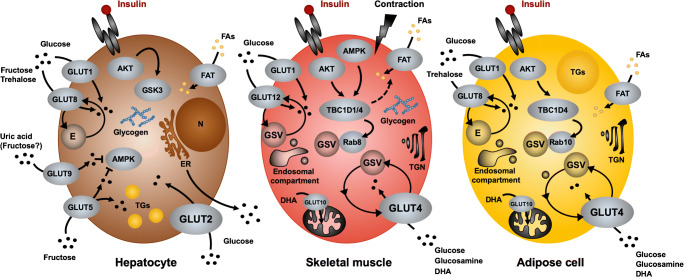
Major facilitative glucose transporters of the GLUT family in the liver, skeletal muscle, and adipose tissue. Several glucose transporters of the SLC4A2 family are involved in cellular uptake of hexoses. Entry of glucose into hepatocytes is mainly catalyzed by the low-affinity, high-capacity GLUT2 transporter which is localized on the cell surface. Following insulin stimulation, glucose is stored as glycogen or released through an ER-dependent mechanism. Other hepatic GLUTs may have accessory functions such as transporting fructose or uric acid. GLUT4 is the principal glucose transporter in adipose and muscle cells and recycles between the plasma membrane and intracellular storage vesicles. Its steady-state distribution is regulated through insulin- and/or contraction-dependent signaling cascades that involve the RabGAP proteins TBC1D1 and TBC1D4. Rab8 and Rab10 have been identified as major GTPases involved in GLUT4 translocation in muscle and fat cells, respectively. In muscle cells, GLUT12 has been described to undergo regulated traffic in response to metabolic stimuli, similar to GLUT4, whereas GLUT8 recycles in adipose cells through endosomal compartments without a known stimulus for translocation. GLUT10 has been shown to facilitate entry of oxidized vitamin C into mitochondria. At least in skeletal muscle, RabGAPs are involved in the regulated entry of fatty acids (FAs) through fatty acid transporters. Arrows indicate flow of substrates, signaling. AKT, protein kinase B; AMPK, 5′ AMP-activated protein kinase; DHA, dehydroascorbic acid; E, endosomal vesicles; ER, endoplasmic reticulum; FAT, fatty acid transporters; GSK3, glycogen synthase kinase 3; GSV, glucose transporter storage vesicles; TGN, *trans*-Golgi network

Gene expression of nearly all GLUTs has been confirmed in the liver. However, as illustrated in Fig. 2, GLUT1, GLUT2, GLUT5, GLUT8, and GLUT9 are particularly abundant in this tissue [109].

#### GLUT1: marker for oncogenic and metabolic diseases in the liver

The facilitative glucose transporter GLUT1 is expressed in most tissues of the body and, due to its low *K*_m_ value for glucose (*K*_m_ = 1–2 mmol/L), is considered as the main GLUT family member regulating basal transport of hexose carbohydrates in a variety of cell types [172]. Highest expression levels have been described for erythrocytes, neuronal membranes, the blood–brain barrier, eye, placenta, and lactating mammary glands. However, GLUT1 also plays a role in the metabolism of liver cells, including both hepatocytes and non-parenchymal cells [108]. Despite GLUT2 being commonly described as the most relevant glucose transporter in the liver, GLUT1 may have a prominent function during early postnatal development [78]. Mice carrying a homozygous knockout for the GLUT1 gene *Scl2a1* are embryonically lethal. Depletion of *Slc2a1* during embryonic development leads to severe malformations of multiple organs, including liver necrosis [89]. Heterozygous *Slc2a1* knockout mice present features of the human GLUT1 deficiency syndrome, a rare metabolic disease characterized by developmental delay and infantile seizures caused by a defective glucose transport across the blood–brain barrier but no metabolic abnormalities [7]. In contrast to hepatocytes that are not strongly depending on external glucose supply, non-parenchymal cells such as endothelial cells and Kupffer cells are not capable of conducting gluconeogenesis and thus rely on glucose uptake rather than endogenous glucose generation. In this cell type, GLUT1 represents the dominant member of the GLUT family [221].

GLUT1 has been implicated in several infectious diseases targeting liver cells. For instance, infection with *Plasmodium berghei*, the parasite causing malaria disease, enhances the translocation of GLUT1 to the cell membrane of hepatoma cells, resulting in significantly increased glucose transport into infected cells [155]. Upon hepatitis C infections, it has been demonstrated that cell surface expression of both GLUT1 and GLUT2 is virally downregulated in hepatocytes, leading to a specific subtype of diabetes. In this context, GLUT2 seems to be regulated at the transcriptional level, whereas GLUT1 membrane localization is impaired due to altered trafficking [111]. A healthy liver expresses only low amounts of GLUT1. In contrast, there is a strong connection between GLUT1 expression and diverse cancer forms. GLUT1 abundance is elevated in hepatocellular carcinoma (HCC), where GLUT1 acts as a tumor promoter and has prognostic and diagnostic significance [267]. Tumor cells demonstrate a substantially enhanced rate of glycolysis, which, in turn, requires increased glucose transport. Upregulation of GLUT1 expression in cancer cells is predominantly mediated by oxygen-related transcription factors, such as the hypoxia-inducible factor 1 (HIF-1) [103].

Moreover, it was shown that expression levels of a number of GLUTs (GLUT1, GLUT3, GLUT5, and GLUT12) are elevated in NASH and alcoholic liver disease (ALD) [109]. Increased expression of GLUT1 can thus be considered as a marker for metabolic and oncogenic diseases in the liver. Interestingly, GLUT1 expression is also increased in hepatocytes in both fasting and diabetic states. It is unclear, however, whether these alterations are triggered rather by low circulating insulin levels or by hyperglycemia [220, 231]. In the context of microvascular complications, however, decreased GLUT1 levels in the retina have been described to be beneficial in the prevention of retinopathy as a diabetic late complication [134]. In addition to circulating glucose or insulin levels, also hypoxia and nitric oxide (NO) have been implicated to stimulate expression levels of GLUT1 in the liver. In turn, oxidative stress and enhanced NO production have been demonstrated to be responsible for the detrimental effects of glucotoxicity. Due to the high glucose affinity of GLUT1, elevated levels of this transporter have been shown to contribute to glucotoxicity by increasing the production of reactive oxygen species (ROS) in the liver [220].

Interestingly, fibroblast growth factor 21 (FGF-21), a circulating factor produced by hepatocytes that has been implicated to act protectively against insulin resistance and type 2 diabetes, mainly by enhancing glucose transport into adipose tissue, stimulates expression levels of hepatic GLUT1 and GLUT4, thereby also increasing glucose influx in an autocrine manner (Fig. 1). In diabetic mice, administration of FGF-21 results in lowered plasma glucose levels, presumably by inhibiting hepatic gluconeogenesis and stimulating glycogen synthesis [130].

#### GLUT2: major glucose transporter required for glucose sensing and hepatic glucose output

Glucose transporter isoform 2 (GLUT2) represents the major member of the GLUT family in pancreatic beta cells and hepatocytes but is also abundant in intestine, kidney, and the central nervous system. Due to its uniquely low affinity for glucose (*K*_m_ ~ 17 mmol/L), GLUT2 plays a crucial role in a variety of glucose-sensing cells, which is sampling a wide range of blood glucose concentrations. In pancreatic beta cells, GLUT2 is required for the control of glucose-stimulated insulin secretion (GSIS). In the central nervous system, more specifically in neurons, astrocytes, and tanycytes, this glucose transporter isoform is involved in the regulation of feeding behavior and thermoregulation as well as in sympathetic and parasympathetic activities [233]. Hepatocytes and beta cells share a common mechanism that translates the response to elevated blood glucose levels to the activation of the transcription factor ChREBP (carbohydrate-response element-binding protein), a key factor inducing glycolytic and lipogenic genes in both cell types [49]. In hepatocytes, GLUT2 controls the majority of glucose uptake dependent on the levels of circulating glucose in the bloodstream (Table 1). Once in the cell, glucose is rapidly phosphorylated to glucose-6-phosphate by the enzyme glucokinase and subsequently metabolized by glycolysis or incorporated into glycogen [99]. In addition to GLUT2, glucokinase is also crucial for maintaining blood glucose levels at a constant concentration of ~ 5 mmol/L (in humans) and genetic mutations in both, GLUT2 and glucokinase, have been associated with disturbances in glycemia and type 2 diabetes [143, 163]. In humans, mutations in the GLUT2-encoding gene *SLC2A2* are associated with glycogen storage defects in kidneys and the liver, and a rare genetic *SLC2A2* deficiency has been established as Fanconi–Bickel syndrome (FBS) which exhibits characteristic features such as hepatomegaly caused by glycogen accumulation, glucose and galactose intolerance, fasting hypoglycemia, tubular nephropathy, and disturbed growth [61, 144]. As a result of this glycogen storage disease (GSD), FBS patients exhibit substantial impairments in whole-body glycemia, more specifically postprandial hyperglycemia and fasting hypoglycemia, both features of an insufficient control of hepatic glycogen metabolism and glucose output [7]. Deficiency in GLUT2 has also been associated with increased urinary excretion of glucose, due to reduced reabsorption of glucose in renal tubular cells [11, 81, 200]. Interestingly, heterozygous knockout mice for the GLUT2 gene *Slc2a2* are metabolically unobtrusive, indicating that GLUT2 abundance is not rate limiting in metabolism [233]. Homozygous whole-body *Slc2a2* knockout mice, in contrast, develop diabetes-like symptoms including hyperglycemia and increased circulating free fatty acid levels early after birth and usually die before weaning age. These mice demonstrate impaired glucose tolerance caused by developmental defects in the α-cell-to-β-cell ratio of the endocrine pancreas [81]. Surprisingly, homozygous *Slc2a2* knockout mice exhibit normal hepatic glucose output, indicating (a) that the liver does not significantly contribute to the observed impairments in glucose tolerance in *Slc2a2*^*−/−*^ mice and (b) that the existence of an alternative signaling pathway is independent from GLUT2 regulating glucose release in hepatocytes [80]. Indeed, *Slc2a2*^*−/−*^ hepatocytes display a fraction of newly synthesized glucose that accumulates intracellularly in the cytosol and is exported via a yet unidentified plasma membrane transport system [95]. In order to overcome the early lethality of GLUT2 deficient mice and to study physiology at later stages, a specific transgenic mouse model overexpressing the GLUT1 gene *Slc2a1* under control of the beta cell–specific rat insulin promoter (RIP) in combination with a global GLUT2 deficiency syndrome (RIP-GLUT1/GLUT2) was generated. In these mice, the primary defect in GSIS caused by the *Slc2a2*^*−/−*^ knockout mice was rescued by a compensatory expression of GLUT1, preventing pre-weaning lethality. RIP-GLUT1/GLUT2 mice display normal postprandial blood glucose levels but fasting hypoglycemia, glycosuria, and an elevated glucagon-to-insulin ratio. The normal glucose tolerance in these mice indicates that GSIS can be restored by GLUT1 as well as by GLUT2 despite the still abnormal composition of the endocrine pancreas [234]. There is evidence for an inter-organ crosstalk between the liver and the endocrine pancreas cells via the hepatoportal glucose sensor. Postprandial stimulation of the vagal afferents within the hepatoportal vein inhibits glucagon secretion from pancreatic alpha cells and, on the other hand, leads to enhanced glucose transport into muscle and adipose tissue [21, 57]. Importantly, induction of hypoglycemia by portal glucose infusion is ablated in RIP-GLUT1/GLUT2 mice, indicating a major role for GLUT2 as a glucose sensor in the hepatoportal vein area, indirectly controlling pancreatic glucagon secretion via the nervous system [20]. A more direct influence of GLUT2 on liver metabolism has been described by studying liver-specific GLUT2 knockout mice. Tamoxifen-induced deletion of GLUT2 specifically in hepatocytes (LG2KO mice) led to the suppression of glucose entry into the liver cells without affecting the glucose output. Whole-body glycemia, however, is unaltered in these mice, presumably due to elevated glucose uptake into the skeletal muscle. Interestingly, GSIS is progressively impaired in LG2KO animals, whereas expression levels of ChREBP and its downstream target genes are increased. In this context, bile acids have been suggested as a mechanistic link between reduced cholesterol biosynthesis genes in the liver and disturbed insulin secretion in beta cells [210].

GLUT2 does not exclusively transport glucose but also other carbohydrates such as galactose, mannose, fructose, and glucosamine [102, 244]. In the recent decade, the impact of a diet high in fructose has raised attention in the context of the obesity epidemic. Like glucose, fructose is transported into liver cells via GLUT2 and subsequently metabolized to glycogen and/or triglycerides. However, unlike glucose, fructose uptake does not trigger insulin secretion in pancreatic beta cells [127]. Enhanced fructose consumption, being the result of a Western diet, leads to elevated accumulation of saturated fatty acids and enhanced gluconeogenesis rates in the liver, eventually inducing liver steatosis. On a molecular level, increased fructose influx into hepatocytes stimulates the expression of lipogenic enzymes such as fatty acid synthase (FAS), stearoyl-CoA desaturase 1 (SCD-1), and acetyl-CoA carboxylase 1 (ACC-1) via activation of the transcription factor ChREBP [101]. The lipogenic features of fructose lead to the development of NAFLD and, as a consequence, to increased hepatic insulin resistance, a disorder worsened by the lower satiety signal derived from fructose metabolism compared to glucose due to the weaker impact on insulin secretion. Compared to a high-fat diet (HFD) containing glucose, fructose-rich HFDs exacerbate the deleterious effects of a Westernized diet on liver function, thereby increasing inflammatory processes, ER stress, and apoptosis [10]. NAFLD represents a major risk factor for the development of liver cirrhosis and is an independent predictor of cardiovascular disease. On a population level, variants in the *SLC2A2* gene have been associated with fasting hyperglycemia, transition to type 2 diabetes, hypercholesterolemia, and risk of cardiovascular diseases in genome-wide association studies (GWAs) [60]. There is evidence that the GLUT2 locus is relevant for the regulation of serum cholesterol levels and increases the risk to develop cardiovascular diseases [18, 98]. It is unclear, however, whether these associations are directly connected to hepatocyte or even beta cell function since there is also a significant impact of GLUT2 on feeding behavior and glucose-regulated autonomic nervous activity in the central nervous system that contribute to the observed metabolic phenotypes. A novel role for liver GLUT2 has been recently proposed during the regulation of circadian rhythm. Interestingly, mice deficient for the *Bmal1* gene, an essential clock gene, demonstrate a disrupted circadian function within hepatocytes with a concomitant decrease in liver GLUT2 abundance. In addition, these mice show fasting hypoglycemia, reduced liver glycogen, and increased glucose clearance, indicating impairments in liver gluconeogenesis [123]. In addition, chronic alcohol consumption disrupts the diurnal rhythm of *Slc2a2* expression in the liver, being accompanied with disturbances in glycogen metabolism [243].

#### GLUT5: main mammalian fructose transporter

In analogy to GLUT2, GLUT5 represents the second relevant GLUT isoform in fructose-mediated development of NAFLD. As already discussed in the above section on GLUT2, high intake of dietary fructose is considered an important contributor to the development of insulin resistance and the metabolic syndrome [266]. The transport activity of GLUT5 is described as specific for fructose, with no ability to transport glucose or galactose. Classically, GLUT5 has been found to be most abundant in both the apical and basolateral membranes of the intestine with a high affinity towards fructose (*K*_m_ = 6 mmol/L) [108]. Interestingly, recent studies have linked dietary fructose consumption with increased hepatic expression of GLUT5, concomitant to an elevated NAFLD development and inflammatory processes [10]. In addition, enhanced expression levels of GLUT5 in the liver due to a high-fructose diet correlated with increased indicators of oxidative stress and mitochondrial dysfunction [5]. GLUT5 knockout mice show massive weight loss and nutrient malabsorption when fed a diet containing fructose but show no impairments under a dietary regimen short of fructose. The observed phenotype of the knockout mice, however, is mainly derived from the intestinal depletion of GLUT5, the liver presumably only playing a minor and secondary role. Of note, GLUT5 is not exclusively responsible for the uptake of hexoses in intestinal cells. In contrast, members of the sodium-dependent glucose cotransporter (SGLT) family of glucose transporters, mainly SGLT1, are the predominant transporters in epithelial cells [254]. Interestingly, *Sglt1*-deficient mice are healthy despite an impaired intestinal glucose absorption when kept on a diet free from glucose and galactose [76]. The classical model of sugar absorption describes that glucose is being actively transported across the brush border membrane whereas fructose crosses the brush border membrane via facilitative diffusion through GLUT5. GLUT2, in contrast, transports glucose from the cytosol to the blood [255]. In summary, GLUT5 is highly relevant for fructose transport in the small intestine but may also contribute to hepatic fructose uptake in hepatocytes.

#### GLUT8: intracellular hexose transporter regulating hepatic oxidative metabolism

The glucose transporter GLUT8 is widely expressed in different glucose-metabolizing tissues such as testis, muscle, brain, liver, and kidney and shows a dual specificity to transport glucose and fructose. Interestingly, GLUT8 shows a reconstitutable glucose transport activity similar to that of GLUT4 [54]. For this reason, it was initially believed that GLUT8 might be the major GLUT isoform compensating for a lack of GLUT4 since early studies of GLUT4 knockout mice demonstrated a substantial growth retardation, decreased longevity, and cardiac hypertrophy but no obvious diabetic phenotype with normal glucose tolerance [112]. However, mice deficient in GLUT8 display unaltered body development and glycemic control, indicating a rather dispensable role in whole-body glucose homeostasis. The main function of this glucose transporter has been determined to regulating energy metabolism of sperm cells [73]. GLUT8 was described as an intracellular hexose transporter with a GLUT4-like translocation activity to the cell surface as response to hormonal stimuli [97]. However, there is no definite conclusion on these trafficking processes since several studies demonstrated that none of the conventional stimuli tested induced a translocation of GLUT8 to the plasma membrane in cultivated cell lines, indicating a predominant role of GLUT8 in catalyzing the transport of sugars or sugar derivatives through intracellular membranes [2, 207]. Nonetheless, there is some evidence in the literature for at least a minor significance of GLUT8 as a cell surface–localized transporter in fructose import into hepatocytes. Whereas GLUT8-deficient mice do not show a pronounced metabolic phenotype when fed a standard chow diet, they display resistance to diet-induced glucose intolerance and dyslipidemia concomitant with enhanced oxygen consumption and thermogenesis when challenged with a high-fructose diet. Apparently, these protective mechanisms are based on elevated abundance of hepatic peroxisome proliferator–activated receptor γ (PPARγ) protein in GLUT8 knockout animals. A direct relation between PPARγ and GLUT8 expression in liver cells was demonstrated by in vivo hepatic adenoviral GLUT8 overexpression that resulted in decreased PPARγ expression levels [43]. In cultured hepatocytes, it was shown that silencing of the GLUT8 gene *Slc2a8* substantially suppresses radiolabeled fructose uptake and de novo lipogenesis. Following a long-term fructose overfeeding, GLUT8 knockout mice display reduced fructose-induced triglyceride and cholesterol accumulation in the liver without changes in hepatic insulin-stimulated Akt phosphorylation [44]. Moreover, during fasting, GLUT8-deficient mice exhibit enhanced thermogenesis, ketogenesis, and peripheral lipid mobilization concomitantly to mildly disturbed hepatic mitochondrial oxidative metabolism in vivo and in vitro. These observations are related to enhanced activation of hepatic peroxisome proliferator-activated receptor α (PPARα) and its transcriptional fasting response target hepatokine, FGF-21. Most importantly, knockdown of PPARα in livers from GLUT8 knockout mice abolishes the elevated ketogenesis and FGF-21 activation, indicating a direct GLUT8-PPARα communication axis [151]. Interestingly, hepatic GLUT8 expression levels are linked to the metabolic state of an organism. Whereas gene expression of *Slc2a8* is reduced in mouse models of autoimmune type 1 diabetes, GLUT8 expression increases in insulin resistance and type 2 diabetes, suggesting that the expression is regulated by insulin. In addition, hepatic GLUT8 expression levels correlate with circulating insulin in diabetic mice, indicating a potential link to whole-body glycemia [77]. In addition to glucose, GLUT8 was described to transport also the disaccharide trehalose, a non-reducing sugar consisting of two molecules of glucose that is mainly found in plants and insects [150]. GLUT8-deficient hepatocytes and GLUT8-deficient mice exposed to trehalose resisted trehalose-induced AMP-activated protein kinase (AMPK) phosphorylation and autophagic induction in vitro and in vivo, indicating a role of GLUT8 in autophagy signaling [150]. While trehalose has been widely used as an experimental inducer of autophagy in cultured mammalian cells, its direct effect on autophagosome formation and autophagy flux has been discussed controversially [125].

#### GLUT9: a high-capacity uric acid transporter compensating for GLUT2

As GLUT8, also GLUT9 belongs to the more recently discovered isoforms of the GLUT family [176]. It is primarily expressed in the liver, kidney, and intestine. Originally described as a hexose transporter, more recent studies could show that the urate transport activity of GLUT9 is 45-fold to 60-fold higher than that of glucose or fructose transport [27]. In this context, a number of GWASs found associations between several variants in the *SLC2A9* gene and serum urate concentrations. Interestingly, these genetic variants were also associated with gout and low-fractional excretion of uric acid (UA) [246]. UA is a product of the purine metabolism and acting as an antioxidant. However, when entering a cell, UA is converted into a pro-oxidant form, increasing cellular oxidative stress and impairing insulin-dependent stimulation of nitric oxide formation [34]. Due to this feature, UA serum levels and their implications on the pathophysiology of the metabolic syndrome and cardiovascular disease (CVD) have been the focus of extensive research throughout the last years [165]. Interestingly, hyperuricemia has been demonstrated to predict the development of diabetes and to mediate the progression of insulin resistance, fatty liver, and dyslipidemia in both fructose-dependent and fructose-independent models of the metabolic syndrome. Novel approaches are currently being tested to improve the prevention of type 2 diabetes or the metabolic syndrome by lowering serum uric acid levels [116]. From studies in GLUT9-deficient mice, it is known that the beneficial effects of lowering serum UA levels may be mainly regulated by enterocytes, since these mice develop impaired enterocyte uric acid transport kinetics, hyperuricemia, hyperuricosuria, spontaneous hypertension, dyslipidemia, and elevated body fat [45]. There is also some evidence for a direct association between the metabolic syndrome and gout pathophysiology. Hyperuricemia represents a key feature of both metabolic diseases by promoting inflammation, hypertension, and cardiovascular as well as liver disease. Relevant in the context of GLUTs, also a diet rich in fructose is associated not just with increased rates of hypertension, weight gain, impaired glucose tolerance, and dyslipidemia but also with an important stimulus of urate biosynthesis. It has been shown that in hepatocytes and other cell types, a fructose/urate metabolic loop leads to the inhibition of AMPK, the AMP-dependent kinase which is crucial in the maintenance of cellular energy metabolism [235]. GLUT9 shows high expression levels in the liver; thus, a role in secreting UA into the circulation has been proposed in humans. Somehow adverse findings, however, have been described in mice, with GLUT9 being responsible to transport uric acid into the liver for further breakdown. Depletion of the *Slc2a9* gene specifically in the liver results in severe hyperuricemia and hyperuricosuria, in the absence of urate nephropathy or any structural abnormality of the kidney as were found in the whole-body knockout model. These data indicate a dual role for GLUT9 in urate handling in the kidney and uptake in the liver [177]. In addition, no direct link between UA and hypertension was found in liver-specific GLUT9 knockout mice [179]. Only when challenged with both a high-fat diet and an inosine gavage, a precursor for UA, did liver-specific GLUT9-deficient mice develop chronic inflammation and acute renal failure [178]. An interesting study analyzing mice that lack GLUT9 specifically in the kidney tubule shows that these animals demonstrate increased excretion of uric acid in the urine (uricosuric effect), associated with reduced plasma urate levels, lower blood pressure, and less renal expression of the kidney injury marker KIM1 [169]. Apart from the indirect impact of hepatic GLUT9 deficiency on kidney function, also a role for this GLUT isoform in hepatocytes has been proposed. As already discussed in the previous section, GLUT2 knockout mice demonstrate unaltered hepatic glucose output, the underlying mechanism still not been understood. There have been controversial reports on the ability of GLUT9 to transport hexoses such as glucose or fructose [8, 13, 141]. The fact that FBS patients display a normal response after fructose administration strongly indicates the presence of an alternative fructose transporter next to GLUT2 in the liver. Due to its high expression levels in this tissue, GLUT9 is still considered a major candidate compensating for the severely impaired hepatic fructose uptake in FBS patients [204].

#### GLUT10: high hepatic expression levels but so far enigmatic function

GLUT10 represents a close homolog of GLUT9 within the GLUT family and is expressed in a variety of tissues such as brain, lung, adipose tissue, heart, placenta, and skeletal muscle, but with highest expression levels in the liver and pancreas. Transport studies in *Xenopus* oocytes revealed GLUT10 transport activity for both glucose and galactose [42, 154]. A contribution of this GLUT isoform to the development of type 2 diabetes is of debate, some GWA studies showing associations of distinct gene variants with diabetes traits, others do not [6, 75, 193]. Despite the relatively high hepatic expression levels, there is no link to date between GLUT10 and liver metabolism. Clear evidence has been gained from murine knockout and clinical studies demonstrating an important role for GLUT10 in arterial diseases. It has been described, for instance, that loss-of-function mutations in the *SLC2A10* gene encoding GLUT10 are responsible for arterial tortuosity syndrome (ATS), a rare congenital connective tissue condition disorder [68].

#### Glucose transporters with minor expression levels or absent in the liver: GLUT3, GLUT4, GLUT6, GLUT7, GLUT11, GLUT12, and GLUT13 (HMIT)

A number of GLUT family members are widely considered as non-relevant in liver metabolism, with expression levels either completely absent or hardly detectable. One of these glucose transporters is GLUT3, a GLUT isoform mainly related to brain metabolism. GLUT3 expression has been described to be restricted to the brain in rodents and being expressed only to minor amounts in the liver in humans [214, 262]. However, in analogy to the GLUT1 expression pattern, also GLUT3 and GLUT5 transporters show increased expression in cancer cells, for instance liver metastatic lesions [121]. An auxiliary function of some GLUTs in the liver seems to be the transport of dehydroascorbic acid (DHA), the oxidized form of ascorbic acid (AA, vitamin C) as described for the GLUT isoforms GLUT1, GLUT3, and GLUT4 [188]. The last-mentioned glucose transporter GLUT4 is known as major isoform in muscular and adipose tissues and only shows minor expression levels in the liver as well [228]. However, GLUT4 deficiency in these organs has been demonstrated to exert secondary impairments of liver insulin sensitivity, mainly due to increased ectopic lipid accumulation in the liver [14]. Expression of GLUT6 has been described for a variety of tissues, including brain, pancreas, and adipose tissue. In the liver, however, this isoform seems to be absent [227]. The GLUT6 gene shows high sequence identity to the GLUT3 gene, and it was speculated that GLUT6 may have emerged by the insertion of the GLUT3 gene into another gene on the same chromosome [113]. GLUT7, in contrast, has been described as a hepatic microsomal GLUT found in the endoplasmic reticulum in the initial reports, mainly being involved in the release of glucose from gluconeogenesis or glycogen breakdown. However, more recent studies demonstrate that this GLUT isoform is essentially not expressed in human or rodent liver cells, assuming that the previous results were due to cloning artifacts [108]. GLUT11 has been described as a transporter for both fructose and glucose in a variety of tissues with at least three different isoforms (GLUT11A, GLUT11B, GLUT11C) specific for distinct cell types, excluding liver cells [72]. GLUT12 is mainly expressed in the skeletal muscle, heart, small intestine, and prostate and has been a candidate to solve the riddle of the normal glucose tolerance in GLUT4-null mice for a while [225]. In the liver, however, this GLUT isoform is not expressed [180]. The same applies to GLUT13 (HMIT), a H^+^-dependent myoinositol cotransporter mainly relevant in the brain [7].

## Skeletal muscle and adipose tissue

### Skeletal muscle is the main tissue controlling postprandial glucose disposal

Skeletal muscle plays a critical role in maintaining blood glucose homeostasis. In fact, skeletal muscle is the major sink for glucose after a meal. The muscle accounts for approx. 75% of glucose disposal following infusion of glucose, and this process is markedly impaired in the insulin-resistant state [47, 48]. Physical exercise increases muscle insulin sensitivity, and both insulin and exercise act synergistically to enhance glucose disposal in skeletal muscle [46]. Both aerobic and resistance exercise training have been shown to lower blood glucose levels which are at least in part due to increased glucose transport activity and glucose metabolism in skeletal muscle. However, the mechanism underlying the beneficial effects of exercise is not fully understood but likely involves alterations in signal transduction and metabolic pathways in multiple organs (Fig. 1).

### Adipose tissue regulates systemic glucose metabolism

Adipose tissue is a highly dynamic organ with a high capacity for remodeling to meet the demands of changing nutritional conditions. Moreover, adipose tissue represents a major endocrine organ that supplies essential hormones and factors controlling whole-body metabolism, systemic insulin sensitivity, and energy homeostasis. Both the absence and excess of adipose tissue may lead to severe impairments of glucose homeostasis and diabetes [133]. White adipose tissue harbors mature adipose cells and precursor cells, but also other cell types related to its innervation and vascularization. Most importantly, it contains various immune cell species that are indispensable for adipocyte function and dynamically adjust to alterations in fat depot size [250]. Adipose cells from different origins, e.g., from subcutaneous or visceral depots, have different metabolic properties and expansion dynamics [82]. In rodents, but also in humans, the brown adipose tissue is specialized to dissipate energy as heat. As a result of these structural complexities, studies on glucose transport in adipose cells usually focus on a specific subset of conditions relevant in adipocyte biology. Adipose tissue plays an important role in glucose and lipid homeostasis, and metabolism of both glucose and lipid is closely intertwined. The contribution of adipose cells to glucose disposal is much smaller compared to skeletal muscle [47, 48]. However, studies using knockout and transgenic mice deficient or overexpressing glucose transporters have demonstrated the critical role of adipose tissue in glucose homeostasis.

### Multiple GLUT isoforms are expressed in skeletal muscle and adipocytes

Skeletal muscle has a profound capacity for taking up glucose from the extracellular medium. While samples from human and rodent skeletal muscle tissue have been found to express multiple glucose transporters belonging to both gene families, GLUTs and SGLTs, the corresponding copy numbers of the respective messenger RNAs (mRNAs) differed over 3 orders of magnitude [227]. These differences might be attributed to the specific skeletal muscle type analyzed or to differences in species and conditions prior tissue sampling. Nevertheless, only a subset of glucose transporters has been detected in skeletal muscle and adipose tissue at the protein level, including GLUT1, GLUT3, GLUT4, GLUT5, GLUT6, GLUT8, GLUT10, GLUT11, and GLUT12. Expression of GLUT isoforms between skeletal muscle and adipose tissue exhibits a substantial overlap (Fig. 2). Table 1 summarizes the metabolic function of the major GLUTs in muscle and fat tissue.

#### GLUT1: major glucose transporter regulating basal glucose transport into skeletal muscle and adipocytes

Skeletal muscle contains GLUT1 mRNA and protein; however, approximately half of the GLUT1 protein in rat skeletal muscle tissue has been attributed to intramuscular nerve cells [85]. In adult skeletal muscle fibers from rodents, GLUT1 protein abundance was found to be fiber type specific, with highest amount in red muscles [106, 146], and increased under conditions during muscle regeneration [71]. GLUT1 has been found primarily localized on the cell surface, suggesting a function in providing glucose transport in the basal state as in many other cell types [85, 146]. However, in several cell types, particularly in tumor cells, a fraction of GLUT1 recycles between internal membrane structures, mostly endosomes, and the plasma membrane. Interestingly, metabolic stress such as hypoxia has been shown to lead to a shift in the distribution of GLUT1 from endosomes to the cell surface through a process which requires the retromer complex and the Rab GTPase–activating protein TBC1D5 [195, 216].

In accordance to skeletal muscle, GLUT1 is also expressed in adipose tissue and in isolated adipose cells albeit at much lower levels compared to GLUT4 [270]. By utilizing an impermeant photoaffinity label, Holman and colleagues [94] found that in adipocytes, insulin leads to translocation of GLUT1 from intracellular vesicles to the plasma membrane, but, to a much lesser extent, compared to GLUT4, i.e., 5-fold vs 20-fold. Cell surface GLUT1 increases also in response to other stimuli, such as phorbol esters, whereas GLUT4 does not, indicating that both transporters are distributed in different types of vesicles. Kinetic analyses showed that insulin-stimulated glucose transport of GLUT1 is rather negligible compared to GLUT4 [94]. Levels of GLUT1 protein are unaffected by diabetes or insulin treatment [105].

#### GLUT3: contributor to basal glucose uptake in skeletal muscle

Human GLUT3 was initially cloned from a fetal skeletal muscle cell line [114], but the protein is predominantly present in neurons [217]. Neuron-specific deletion of the GLUT3 gene *Slc2a3* leads to distinct postnatal and adult neurobehavioral phenotypes [215]. GLUT3 protein was found in human gastrocnemius muscle samples from autopsies and in cultured rat L6 muscle cells [15, 226]. The exact fiber-type localization of GLUT3 has not been reported, and its relatively low *K*_m_ value for glucose (1.4 mmol/L) may suggest a role in basal glucose uptake in skeletal muscle [245]. Interestingly, GLUT3 strongly increased during cell differentiation of rat myoblasts to myotubes and was reduced after muscle cell contraction. Moreover, stimulation of L6 cells with insulin and IGF-I was shown to increase cell surface expression of GLUT3 [15] whereas stimulation with triiodothyronine (T_3_) increased total GLUT3 but not cell surface expression of the transporter GLUT3 content [232]. The role of GLUT3 in skeletal muscle remains elusive. GLUT3 is not present in adipose tissue [245].

#### GLUT4: the workhorse for insulin- and contraction-responsive glucose transports in skeletal muscle and adipocytes

GLUT4 is the most abundant glucose transporter in skeletal muscle [50] and has been considered to be rate limiting for glucose uptake and metabolism, at least in the resting state of the muscle [131, 148]. Muscle-specific knockout of GLUT4 in mice led to systemic insulin resistance and a mild diabetic phenotype [115] whereas overexpression of GLUT4 improved glucose tolerance and insulin sensitivity in normal as well as genetically diabetic db/db mice [19, 237]. In isolated skeletal muscle, overexpression of GLUT4 increased insulin-stimulated glucose transport activity [86] whereas GLUT4 ablation was found to reduce insulin-stimulated glucose uptake [222]. These findings indicate a central role of GLUT4 in whole-body metabolism and glucose uptake in skeletal muscle (Table 1).

Following the initial proposal of the “translocation hypothesis,” it is now well established that GLUT4 undergoes a rapid and reversible translocation from intracellular compartments to the cell surface [38, 229]. In non-stimulated skeletal muscle and adipose cells, GLUT4 resides in specialized intracellular storage vesicles (glucose transporter storage vesicles, GSVs) and is slowly but constantly recycling between this compartment and the plasma membrane (Fig. 2). Internalization and subsequent sorting of GLUT4 requires interaction of specific intracellular residues in GLUT4 with clathrin adaptor proteins [4]. Consequently, blocking the endocytosis by overexpression of a dominant-negative mutant of the GTPase dynamin leads to accumulation of GLUT4 on the cell surface in the basal state [3, 107]. Using a membrane-impermeable photolabel, Satoh and colleagues [205] demonstrated that insulin markedly accelerates the exocytosis of GLUT4-containing vesicles, leading to a rapid and reversible redistribution of GLUT4 from GSVs to the PM and, subsequently, to increased influx of glucose into the cells. Importantly, in skeletal muscle, exercise and muscle contraction also lead to translocation of GLUT4 to the cell surface [58, 67]. Both insulin- and contraction-stimulated translocations are additive, and it has been proposed that both stimuli utilize distinct intracellular GLUT4 storage pools [59]. Several signaling pathways have been implicated to play roles in regulating GLUT4 translocation in response to insulin and contraction [62, 100, 118, 187].

GLUT4 has a *K*_m_ value for glucose of about 5 mmol/L [197], close to blood glucose levels in healthy human individuals. Glucose that is transported into skeletal muscle and adipocytes is trapped in the cell as glucose-6-phosphate after phosphorylation by hexokinase. Among several metabolic pathways utilizing glucose, the glycogen synthesis pathway is highly significant in skeletal muscle as it provides the most relevant energy storage form for this tissue. In fact, muscle-specific knockout of glycogen synthase greatly diminishes glycogen stores and exercise performance [259] whereas overexpression has the opposite effect on glycogen stores [140]. Consistent with the rate-limiting role of GLUT4 in glucose metabolism, overexpression of GLUT4 in muscle leads to increased glycogen stores in the insulin-stimulated state [237]. However, despite strongly reduced insulin-stimulated glucose uptake in muscle-specific GLUT4 knockout mice, muscle glycogen levels are normal or even increased in the fasted state [115], indicating possible compensatory mechanisms for glucose import.

GLUT4 is the most abundant glucose transporter in adipose cells [104]. Transgenic mice expressing high levels of GLUT4 in adipose tissue are highly insulin sensitive and glucose tolerant [213]. Adipose-specific GLUT4 knockout mice had normal adiposity but whole-body glucose intolerance and insulin resistance [1], indicating the critical role of adipose GLUT4 in systemic glucose homeostasis and organ crosstalk (see below). In type 2 diabetes, GLUT4 expression in adipose tissue is substantially downregulated but unaltered in skeletal muscle [69, 219].

GLUT4 also transports glucosamine with a *K*_m_ value of ~ 4 mmol/L [244] and DHA with a *K*_m_ value of ~ 1 mmol/L [197]. Glucosamine is a specific precursor of β-*N*-acetylglucosamine (GlcNAc) which is required for glycosylation of proteins and thus a major carbohydrate component of many glycoproteins. Specifically, β-*N*-acetylglucosamine (*O*-GlcNAc) represents a regulatory posttranslational modification of nuclear and cytosolic proteins to regulate cell signaling pathways and protein activity similar to phosphorylation. Both elevated flux through the hexosamine biosynthetic pathway and increased *O*-GlcNAc modification of insulin signaling proteins were found to be associated with insulin resistance and impaired GLUT4 translocation in response to insulin in muscle and fat tissue [35]. High concentrations of glucosamine (millimolar range) were shown to inhibit glucose uptake in cultured myotubes in vitro, presumably due to induction of ER stress [182, 190]. On the other hand, glucosamine was shown to extend the life span of *Caenorhabditis elegans* and aging mice which was associated with an induction of mitochondrial biogenesis, lowered blood glucose levels, and increased amino acid catabolism, as found in the context of low-carbohydrate diets [251]. Interestingly, a recent study showed that long-term (8-year) supplementation of glucosamine is associated with a lower risk of incident type 2 diabetes in humans [137].

The GLUT family of transporters may constitute the main entry route for glucosamine into the cell, and both GLUT1 and GLUT4 have been shown to transport glucosamine with similar kinetics [244]. However, as glucosamine is mainly produced endogenously from glucose via fructose-6-phosphate through the hexosamine biosynthesis pathway and glucosamine concentrations in the blood typically do not exceed 0.1 mmol/L [209], i.e., 10-fold below the *K*_m_ value of the GLUTs, it remains to be established whether and how GLUTs contribute to glucosamine-mediated systemic effects on insulin sensitivity in skeletal muscle and adipose tissue.

GLUT4 like GLUT1 and GLUT3 transports DHA, the oxidized form of ascorbate or vitamin C with *K*_m_ values of about 1.5 mmol/L, respectively [197]. In humans, the majority of intestinal vitamin C uptake depends on sodium-dependent vitamin C transporters belonging to the SVCT family of proteins that actively cotransport sodium ions and ascorbate across membranes [240]. Ascorbate serves as an electron donor in many biological redox reactions and constitutes an important part of the cellular antioxidant defense. Oxidation of ascorbate subsequently results in formation of dehydroascorbic acid which is then quickly reduced back to ascorbate [136]. In healthy individuals, plasma concentrations of DHA are in the lower micromolar range, about 10 times less than ascorbate [135]. This has led to the conclusion that glucose transporter–mediated DHA transport may not have a substantial effect on the distribution of DHA and ascorbate under normal conditions [136]. In vitro, glucose inhibits transport of dehydroascorbic acid into red blood cells, and it was shown that in hyperglycemia and diabetes, ascorbate concentrations in human red blood cells were reduced, associated with impairments in cell structure [241, 242]. As GLUT4 is the main glucose transporter in skeletal muscle, it remains to be established whether an impaired DHA transport into skeletal muscle in insulin resistance may contribute to the tissue-specific pathology of diabetes.

Control of GLUT4 expression in skeletal muscle appears to be highly conserved across species [147]. Regulatory sequences required for tissue-specific expression of GLUT4 in skeletal muscle have been mapped to a 1.1-kbp segment in the 5′ region of the GLUT4 gene [171]. Several factors including myocyte enhancer factor 2A (MEF2A) and glucose enhancer factor (GEF) were shown to bind as a complex and synergistically increase GLUT4 promoter activity [119]. Other factors suggested to be involved in the transcriptional regulation of the GLUT4 gene include SP1, CCAAT/enhancer-binding protein (C/EBP), PPARγ, hypoxia-inducible factor 1α (HIF-1α), E-box, sterol regulatory element–binding protein 1c (SREBP-1c), Krüppel-like factor 15 (Klf15), and nuclear factor 1 (NF1) [110, 269]. In addition, histone deacetylase 5 (HDAC5) has been implicated in the regulation of the *Slc2a4* promoter in skeletal muscle, in particular in response to exercise, where nuclear localization of HDAC5 decreases the expression of GLUT4 [152, 167]. Expression of GLUT4 in muscle is upregulated in response to exercise [185] and greatly decreased after muscle immobilization atrophy [51]. Likewise, denervation rapidly reduces the abundance of GLUT4 and leads to a compensatory increase in GLUT1 [16], indicating the importance of electromyogenic, contractile, neuronal, and/or metabolic signals in maintenance of glucose transporter expression patterns [187].

Importantly, isolation of primary rat adipocytes is associated with a rapid decrease (20-fold) in GLUT4 mRNA levels with a concomitant increase (70-fold) in GLUT1 mRNA levels within 24 h, further emphasizing the importance of extracellular signal for GLUT homeostasis [74]. While insulin resistance and obesity are associated with downregulation of GLUT4 expression in adipose tissue [69, 219], GLUT4 levels in diabetic skeletal muscle are mostly unchanged [174]. Likewise, chronic fasting reduces GLUT4 expression in adipose tissue but has little effect on GLUT4 mRNA in skeletal muscle [30]. Several microRNAs have been identified that affect GLUT4 expression and may be altered in the diabetic state, including miR-21a-5p, miR-29a-3p, miR-29c-3p, miR-93-5p, miR-106b-5p, miR-133a-3p, miR-133b-3p, miR-222-3p, and miR-223-3p [63]. Likewise, miRNAs may also regulate the expression of genes that are important for the translocation machinery of GLUT4 in muscle and adipose cells, thus having a direct effect on glucose uptake in these tissues.

#### GLUT8: intracellular transporter with links to developmental insulin signaling and autophagy

GLUT8 represents a high-affinity (*K*_m_ 2 mM) glucose transporter present in specific areas of the brain and other tissues including testis, skeletal muscle, adipose tissue, and liver [73]. Like GLUT1 and GLUT4, GLUT8 transports glucose with a *K*_m_ value of about 2 mmol/L [207] as well as oxidized vitamin C (DHA) with a *K*_m_ value of approx. 3 mmol/L [37]. It also transports the disaccharide trehalose [150]. Interestingly, GLUT8 was reported to undergo insulin-stimulated translocation to the cell surface in the mouse blastocyst [23] but not adipose cells [128]. While a study failed to detect GLUT8 protein in human skeletal muscle [72], others found the protein present in equine skeletal muscle where it was increased in response to 5-aminoimidazole-4-carboxamide ribonucleotide (AICAR), an AMPK activator and putative exercise mimetic [156]. Targeted disruption of *Slc2a8* in mice did not alter glucose and energy metabolism, indicating that GLUT8 does not play a major role for maintenance of whole-body glucose homeostasis, at least in the absence of a metabolic challenge [73].

GLUT8 protein has been detected in adipose tissue of adult mice, albeit at relatively low levels compared to blastocysts, suggesting a function of the transporter in embryonal tissue [23]. In fact, GLUT8 expression increases markedly during fat cell differentiation [206]. The transporter carries an N-terminal dileucine targeting motif that confers intracellular sequestration of the protein in all cells analyzed [207]. In adipose cells, GLUT8 recycles in a dynamin-dependent manner between internal membranes of endosomal origin [9] and the plasma membrane in rat adipose cells, but is unresponsive to stimuli that induce translocation of GLUT4 [128]. In contrast, insulin was reported to cause the expression of the protein on the cell surface of mouse blastocysts which points to a role of this transporter in developmental biology [23]. Interestingly, GLUT8 was found to be required for trehalose-induced autophagy in the liver that is associated with activation of AMPK [150]. Induction of autophagy by both trehalose and physical exercise let to increased expression of GLUT8 in the brain of adult mice [164]. These findings may suggest a specific function of GLUT8 in cellular energy sensing under conditions of energy deprivation.

#### GLUT10: enigmatic glucose transporter also expressed in skeletal muscle and adipose tissue

GLUT10 was initially identified as a high-affinity glucose transporter (*K*_m_ 0.3 mmol/L for glucose) present in various human tissues including brain, liver, heart, skeletal muscle, and pancreas [42]. Interestingly, in smooth muscle cells, GLUT10 was found to localize predominantly to mitochondria where it facilitates transport of l-dehydroascorbic acid (DHA), the oxidized form of vitamin C, into the organelle. As a result, it was suggested that GLUT10 may be part of a protective mechanism of mitochondria against oxidative stress [126]. In mice, chronic muscle loading resulted in an approx. 2-fold increase in protein [153]. However, a possible role of GLUT10 in metabolism remains to be investigated.

GLUT10 was reported to be expressed in cultured murine adipocytes. It was shown to primarily localize to the Golgi apparatus under basal conditions where it translocated to mitochondria upon insulin stimulation [126]. Insulin stimulation increased the influx of DHA into mitochondria where it may play a role in protection from oxidative stress by reducing ROS production [126]. Genetic studies did not find an association with diabetes-related traits in humans [6, 193]. Thus, the function of GLUT10 in glucose homeostasis remains to be clarified.

#### GLUT11: fructose transporter specific for muscular tissues

GLUT11 is closely related to the fructose transporter GLUT5 and is expressed in various tissues, most abundantly in skeletal muscle and the heart [53]. Three splice isoforms were described on both mRNA and protein levels [53, 257]. The glucose transport activity of GLUT11 was markedly inhibited by fructose [53]. In biopsies of human skeletal muscle, immunohistochemical analysis localized GLUT11 exclusively to slow-twitch muscle fibers [72]. Abundance of GLUT11 was unchanged under physiological and pathophysiological conditions including obesity and diabetes [72]. Both substrate specificity and function of GLUT11 in skeletal muscle remain unknown.

#### GLUT12: compensatory glucose transporter upon GLUT4 deficiency in skeletal muscle

GLUT12 is predominantly expressed in insulin-sensitive tissues such as heart, liver, fat, and skeletal muscle. In *Xenopus* oocytes, GLUT12 prefers glucose over fructose and galactose as a substrate [191]. Interestingly, glucose transport was stimulated by sodium ions, indicating an electrogenic Na^+^/glucose symport of GLUT12 [191]. However, the exact substrate specificity and the kinetic constants have not been determined yet.

GLUT12 has received much attention as a possible alternative glucose transporter to GLUT4 [180] as GLUT4 knockout mice showed some residual insulin–stimulated glucose uptake in isolated soleus muscle from female animals [222]. In fact, fractionation experiments demonstrated insulin-stimulated translocation of GLUT12 from intracellular compartments to the plasma membrane in human muscle biopsies and cultured rat L6 myoblasts [225]. Moreover, inhibition of phosphoinositide-3 kinase (*PI3K*) *with the* inhibitor LY294002 prevented translocation of both GLUT4 and GLUT12 in response to insulin, suggesting a similar mechanism involved in the signaling cascade. Transgenic mice that overexpress GLUT12 globally under the control of a beta-actin promoter exhibited increased glucose tolerance and improved whole-body insulin sensitivity [181]. The level of protein overexpression in white adipose tissue, skeletal muscle, and liver of the transgenics was approximately 50% above that of GLUT12 in wild-type littermates. It is therefore difficult to estimate the contribution of endogenous GLUT12 to whole-body glycemic control. Nevertheless, in humans, intensive exercise training (6 weeks of cycling) was reported to increase the abundance of GLUT12 protein in vastus lateralis muscle by a factor of 2, implicating that GLUT12-mediated glucose transport in skeletal muscle might be of physiological relevance, at least under trained conditions [224].

Interestingly, a recent report suggested that GLUT12 may act as insulin-responsive glucose transporter in skeletal muscle of chicken that naturally lack GLUT4 but show a moderate insulin-stimulated glucose disposal into muscle after injection of insulin [236]. No insulin-stimulated glucose transport was observed in cardiac muscle or adipose tissue. As such, GLUT12 might be part of a conserved avian glucose transport mechanism specifically acting in skeletal muscle.

#### Glucose transporters with minor abundance or absent in skeletal muscle and adipocytes: GLUT2, GLUT5, GLUT6, GLUT7, GLUT9, and GLUT13 (HMIT)

GLUT5 is a transporter for fructose but not glucose [113, 149] and is present predominantly in the small intestine where it is required for intestinal fructose absorption [113, 149]. GLUT5 protein was also detected in skeletal muscle from rats and humans [39, 96]. However, as the *K*_m_ value of GLUT5 for fructose (*K*_m_ ~ 6–8 mM) [96] is well above (> 10-fold) postprandial fructose concentrations in the circulation even after a sucrose load, it remains unclear whether this transporter contributes to hexose uptake in muscle. GLUT6 is a rather poor glucose transporter expressed mainly in the brain, spleen, and peripheral leucocytes. GLUT6 has been characterized as having low affinity for glucose, the substrate preference is unknown [52]. In rat adipose cells, GLUT6 was shown to recycle in a dynamin-dependent but insulin-independent manner between vesicles and the plasma membrane [128]. The protein was found to be increased substantially (> 3-fold) in mouse skeletal muscle after chronic muscle loading [153]. However, CRISPR/Cas9–mediated deletion of *Slc2a6* did not alter glucose tolerance, blood glucose, and insulin levels in mice [22]. Thus, in rodents, GLUT6 may not have a major role in regulating metabolism, at least in the sedentary state. GLUT2, GLUT7, GLUT9, and GLUT13 are not expressed in muscle [227].

### In skeletal muscle and adipose cells, RabGAPs relay insulin/contraction signaling to the GLUT4 translocation machinery

In fat and muscle cells, the steady-state distribution of GLUT4 between intracellular compartments and the cell surface is acutely regulated by a complex cascade of phosphorylation events downstream of the insulin receptor [100, 118]. Of the more than 60 known 21-kDa Rab GTPases in mammals, several members of this family including Rab4, Rab5, Rab8a, Rab10, Rab11, Rab13, Rab14, Rab28, and Rab35 have been implicated to play roles in GLUT4 vesicle traffic [100]. In fact, Rab GTPases are considered master regulators of membrane traffic that interact with effector proteins and contribute to membrane tethering events during vesicle transport. Rab GTPases cycle between the GTP-bound form, thought to represent the active state, and the inactive GDP-bound form. The conversion between the two states, GTP-bound and GDP-bound, is catalyzed by Rab GTPase–activating (GAP) proteins and guanine-nucleotide exchange factors (GEFs) that accelerate the dissociation of GDP and reloading of the Rabs with GTP [265]. Several lines of evidence indicate that the two related RabGAPs, TBC1D1 and TBC1D4, are playing critical roles in intracellular sorting and translocation of GLUT4 to the plasma membrane in response to insulin and contraction, the latter being relevant in skeletal muscle [199]. *TBC1D1* is predominantly expressed in skeletal muscle whereas *TBC1D4* is expressed in both skeletal muscle and adipose tissue. Both TBC1D1 and TBC1D4, also known as AS160, are substrates for AKT kinase and other protein serine/threonine kinases including AMPK [199]. In adipocytes and muscular tissue, Rab8a, Rab10, and Rab14, all substrates for TBC1D1 and TBC1D4 in vitro, are associated with GLUT4 storage vesicles [158, 184, 189]. While the exact function of the RabGAPs in the different steps of GLUT4 translocation is not fully understood, mutational analyses indicate that TBC1D1 and TBC1D4 exert an inhibitory effect on GLUT4 translocation that is relieved by phosphorylation at specific residues [138]. Overexpression of phosphorylation-defective mutants of the RabGAPs reduced insulin-dependent GLUT4 translocation, and conversely, deletion of TBC1D1 or TBC1D4 elevated the proportion of GLUT4 protein in the plasma membrane in the absence of insulin stimulation [28]. TBC1D1 is phosphorylated by AKT at Ser^231^ and Thr^590^, whereas TBC1D4 has at least six phosphorylation motifs for AKT [139]. In response to muscle contraction, AMPK has been described to phosphorylate at least 5 to 7 sites in TBC1D1 and TBC1D4, respectively [62]. Current research investigates the contribution of the individual phosphorylation sites in the RabGAPs and their possible interactions with effectors.

The TBCD1 family of RabGAPs comprises more than 30 members that are likely involved in various vesicle trafficking steps. In addition to the more thoroughly studied TBC1D1 and TBC1D4, two additional RabGAPs (TBC1D13 and TBC1D15) have been linked to GLUT4 vesicle traffic by acting on Rab35 and Rab7, respectively [41, 256]. While TBC1D1 and TBC1D4 contain PTB domains that are required for targeting of the proteins to GLUT4 vesicles [138, 139, 183], TBC1D13 and TBC1D15 do not contain such annotated domains and it remains to be established if and how these GAPs are acutely regulated, and at which step they contribute to GLUT4 sorting.

Proteins of the DENN (differentially expressed in normal and neoplastic cells) domain containing family function as Rab-specific GAPs [145, 264]. Of the 18 known members, the Rab10-specific DENND4A, DENND4B, and in particular, DENND4C were shown to inhibit insulin-stimulated GLUT4 translocation upon knockdown in cultured 3T3-L1 adipocytes [203]. In contrast, knockdown of Rabin8, a GEF specific for the TBC1D1/4 substrate Rab8 did not inhibit GLUT4 translocation which might indicate cell type specificity of Rab action. Nevertheless, it remains unknown whether and how the regulation of the GEF activity is linked to insulin signaling. Adding to the complexity, it has been suggested that Rabs, GAPs, and GEFs act in concert by forming cascading networks that regulate membrane flow [168].

TBC1D4 may not be exclusively involved in GLUT4 vesicle traffic as it has been recently shown to participate in the cell surface expression of GLUT12 in response to activation of calcium/calmodulin-dependent protein kinase kinase 2 (CaMKK2) and AMPK signaling [253]. Likewise, overexpression of phospho-site mutants of TBC1D1 and TBC1D4 was reported to reduce cell surface expression of GLUT1 in non-insulin target cells [90]. Interestingly, knockdown of TBC1D5 increases GLUT1 translocation to the plasma membrane, presumably through altered retromer recruitment [195]. These findings underscore an important role of RabGAPs in determining the subcellular distributions of GLUTs between different membrane compartments. Not surprisingly though, several studies indicate that TBC1D GAPs also participate in a variety of other trafficking processes such as retromer-mediated retrograde transport from endosomes to the Golgi [208], synaptic vesicle recycling [211], autophagosome formation [132], and intracellular trafficking of vesicles destined for cell surface antigen presentation [252].

### TBC1D1 and TBC1D4 are associated with metabolic traits and diseases

Mutations in *TBC1D1* have been associated with obesity-related traits in human [157, 223, 247] and mice [29, 55, 88]. In addition, mutations in *TBC1D4* have been linked with insulin resistance in humans [40]. Importantly, a common loss-of-function mutation in *TBC1D4* (p.Arg684Ter) has been recently discovered in the Greenlandic Inuit population where the homozygous carriers of the mutant allele show severely impaired postprandial disposal of glucose and a more than 10-fold increased risk of developing type 2 diabetes [159]. In fact, *TBC1D4* (p.Arg684Ter) appears to be the major genetic cause for type 2 diabetes in both the Greenlandic and Canadian Inuit [142]. Deficiency in TBC1D4 is highly associated with substantially reduced abundance (up to 50%) of GLUT4 in skeletal muscle and adipose cells whereas levels of other GLUTs (GLUT1 and GLUT12) are unaltered [28]. Consequently, *Tbc1d1* knockout mice exhibit severely reduced insulin-stimulated glucose uptake in skeletal muscle whereas *Tbc1d4* knockout animals show blunted glucose uptake in skeletal muscle and adipose cell after insulin stimulation [28, 88]. Because *Slc2a4* mRNA levels are unaltered, the reduced amount of GLUT4 is best explained by missorting and posttranslational degradation of the protein [28]. Nevertheless, deficiency in only one of the two RabGAPs in mice leads to rather moderate impairments in systemic insulin sensitivity and glucose tolerance, indicating a possible compensatory function of the other respective isoforms [29, 56, 124, 249]. Furthermore, the reduction of GLUT4 observed in the single RabGAP knockouts is not higher than in the *Tbc1d1*/*Tbc1d4*-double knockout, indicating that critical sorting steps for GLUT4 are only in part dependent on the two RabGAPs.

### Physical exercise improves glycemic control through enhancing glucose transport

Exercise training increased whole-body insulin-mediated glucose disposal in obese type 2 diabetic patients, and these changes are associated with increased GLUT4 protein content in skeletal muscle [170]. Furthermore, the increased muscle insulin sensitivity of glucose transport after exercise has been shown to result from enhanced translocation of GLUT4 to the cell surface independent of insulin signaling [87]. Exercise and contraction were shown to substantially increase glucose transport in skeletal muscle of wild-type mice but not in GLUT4 knockout mice, indicating the fundamental role of GLUT4 in this tissue [198]. Interestingly, in humans, intensive exercise training (6 weeks of cycling) was reported to increase the abundance of GLUT12 protein in vastus lateralis muscle by a factor of 2, implicating that GLUT12-mediated glucose transport in skeletal muscle might be of physiological relevance, at least under trained conditions [224]. In addition to improvements in skeletal muscle glucose transport [64, 187], exercise has profound beneficial effects on insulin sensitivity at many different sites of insulin action, in particular in the insulin-resistant and diabetic state [192].

Exercise training was found to enhance insulin-stimulated glucose uptake in skeletal muscle and whole-body insulin sensitivity in an AMPK-dependent manner in both healthy and insulin-resistant states [24, 26, 117, 186]. A recent study demonstrated that activation of AMPK leads to enhanced phosphorylation of TBC1D4 at Thr^649^ and Ser^711^ in response to insulin, indicating that RabGAPs may integrate signals from different cellular energy sensors [117].

### Role of glucose transporters in intra-organ crosstalk

Homozygous mice with the GLUT4-null allele displayed a less severe metabolic phenotype than heterozygous global knockout animals with reduced abundance of GLUT4 in adipose tissue and skeletal muscle [194, 222]. This was attributed to compensatory mechanisms that are not yet understood but may allow survival. However, conditional deletion of GLUT4 in either adipose tissue or skeletal muscle causes systemic insulin resistance and results in profound metabolic effects on other tissues. Muscle-specific GLUT4 deficiency decreased insulin sensitivity in adipose tissue and liver [268], whereas adipose-specific GLUT4 deletion leads to insulin resistance in the liver and skeletal muscle [1]. It should be noted that glucose transport in adipose cells contributes rather little to whole-body glucose disposal compared to skeletal muscle. Overexpression of GLUT4 in adipose tissue (aP2 promoter driven) led to a reversal of whole-body insulin resistance in muscle-specific GLUT4 knockout mice, however, without restoring glucose transport in skeletal muscle [25]. Collectively, these findings implicate a complex network by which glucose sensing through GLUT4 in muscle and fat cells may operate to integrate whole-body energy metabolism (Fig. 1). While the details of these circuits are not completely understood, a few circulating molecules including retinol (vitamin A) binding protein 4 (RBP4), branched fatty acid esters of hydroxy fatty acids (FAHFAs), and transforming growth factor β2 (TGF-β2) have emerged in recent years that may play important roles in inter-organ communication [104, 230].

RBP4 is a lipocalin family protein that binds lipid compounds such as fatty acids, steroids, and bilins in the blood. RBP4 is secreted from GLUT4-deficient adipose tissue [261] in mice and elevated in the serum of insulin-resistant and diabetic subjects, as well as in first-degree relatives with a high risk of developing diabetes [79]. In fact, RBP4 secretion inversely correlates with systemic insulin sensitivity. The diabetogenic effect of RBP4 has been attributed at least in part its propensity to activate monocytes, macrophages, and dendritic cells in adipose tissue that might drive adipose inflammation and systemic insulin resistance [161]. A recently discovered lipid species, branched FAHFAs, is also released from adipose tissue, and its levels are highly correlated with insulin sensitivity [263]. FAHFAs have beneficial metabolic effects, including enhancing insulin-stimulated glucose transport and glucose-stimulated GLP1 and insulin secretion, as well as powerful anti-inflammatory properties. It has been shown that GLUT4 and adipose tissue glucose uptake induce and activate the nuclear transcription factor ChREBP, which enhances lipogenesis and the synthesis of these FAHFAs [91, 160].

TGF-β2 is a cytokine secreted from adipose tissue in response to exercise and improves glucose tolerance in mice. Lactate which is released from skeletal muscle during exercise stimulates gene expression of TGF-β2 also in human adipocytes (Fig. 1). TGF-β2 stimulated glucose uptake in cultured muscle cells and adipocytes, brown adipocytes, and oxidative skeletal muscle fibers, but not in glycolytic skeletal muscle through signaling of the TGF-beta receptor [230]. In addition to enhancing glucose uptake, TGF-β2 substantially increased fatty acid uptake and oxidation in cultured adipocytes and skeletal muscle cells. While the mechanism of action is not entirely clear, the beneficial effect of TGF-β2 on glucose metabolism has been attributed in part to its actions as an immune suppressor in adipose tissue [230]. TGF-β2 improved glycemic control also in obese, high-fat diet-fed mice, and it will be interesting to investigate these findings in other genetic models and individuals with type 2 diabetes undergoing exercise training.

Another recent study has demonstrated that serum from healthy subjects that conducted 60 min of cycling shows increased GLUT4 expression in cultured adipocytes [66]. While the source tissue of this circulating factor is unknown, it becomes evident that acute exercise has remote effects on glucose transport effectors in different tissues.

Thus, while GLUT4 in muscle and adipose tissue is clearly indispensable for normal systemic glucose homeostasis, it may constitute part of an important glucose sensor system in the adipose tissue to achieve homeostasis in energy metabolism through regulation of insulin sensitivity in other cell types.

### The etiology of insulin resistance is unknown

Insulin resistance and type 2 diabetes are associated with impaired insulin-stimulated glucose uptake in skeletal muscle and adipose tissue. In mice, overexpression of GLUT4 but not GLUT1 in skeletal muscle normalizes insulin sensitivity and glucose tolerance, indicating that GLUT4 translocation is essential for glycemic homeostasis [148, 237–239]. However, the causal molecular mechanisms for the reduction in insulin action are not fully understood. Alterations in lipid metabolism and production of toxic metabolites, e.g., DAGs [202], ceramides [31], and ROS [166], as well as inflammation [201] have been proposed to inhibit insulin signaling towards GLUT4 through interference with phosphorylation events at the level of the insulin receptor (IR), insulin receptor substrate 1 (IRS1), and downstream effectors. However, this concept has been challenged recently, as experimental insulin resistance can occur independent of alterations in IR and IRS1 signaling [65]. Interestingly, despite possibly shared signaling pathways via RabGAPs, contraction-induced GLUT4 translocation in skeletal muscle is normal under conditions of insulin resistance, suggesting that specific pathways regulating GLUT4 translocation may be intact even in the diabetic state [120]. Deletion of both RabGAPs, however, impairs GLUT4 traffic, thus affecting the insulin-sensitive and contraction-sensitive pathways to a similar degree [28]. In addition to signaling defects, compromised insulin action may also include sorting of GLUT4 through multiple membrane compartments, docking, and fusion of membranes. In addition to signaling events, secondary modifications of GLUT4 and associated sorting proteins may also be compromised in insulin-resistant states, such as ubiquitinylation [122], SUMOylation [129], *N*- and *O*-glycosylation [35, 92], and possibly others. Interestingly, oxidative stress has been linked to carbonylation and oxidation-induced inactivation of GLUT4 in response to diet-induced obesity [17]. Collectively, insulin resistance and diabetes are associated with profound alterations in cellular glucose transport, but the cause and consequence of impaired insulin-stimulated glucose transport in the pathogenesis of the disease remains to be further investigated.

## Conclusion

Previous research has successfully identified a large number of different GLUT isoforms in the liver, skeletal muscle, and adipose tissue (Fig. 1). However, the high degree of substrate variability, complex expressional regulation, and activity patterns of the distinct isoforms indicates that there is much more to unravel. In particular, the influence of different lifestyle factors such as high-fructose diets and exercise on GLUT function in energy metabolism may present a fascinating research area also in the future. GLUT4 remains to be the workhorse for the insulin-regulated glucose transport in adipose cells and for insulin- and contraction-stimulated glucose uptake in skeletal muscle. Numerous studies have established the view that impaired GLUT4 translocation is a critical contributor in the etiology of insulin resistance and type 2 diabetes. The mechanistic framework for this process is exceedingly complex and will likely be a hot topic for years to come. Nevertheless, in addition to GLUT4, other non-classical GLUTs such as GLUT12 may also play roles in fine-tuning glucose uptake and substrate metabolism in insulin-sensitive tissues in response to different physiological cues and/or increased energy demand (Fig. 2, Table 1). Furthermore, other GLUTs such as GLUT8 may provide inducible glucose transport capacity during different stages in cellular development and thus could contribute to the development of insulin resistance at early stages of life. Despite their annotation, several glucose transporters such as GLUT6, GLUT10, and GLUT11 may not be relevant for hexose transport at all, as exemplified by the uric acid transporter GLUT9. Understanding the complex relationship of these metabolic networks and organ crosstalk will represent a fundamental component in the challenge to oppose metabolic diseases.
